# CRISPR-Cas9 Arabidopsis mutants of genes for ARPC1 and ARPC3 subunits of ARP2/3 complex reveal differential roles of complex subunits

**DOI:** 10.1038/s41598-022-22982-8

**Published:** 2022-10-28

**Authors:** Erica Bellinvia, Judith García-González, Petra Cifrová, Jan Martinek, Lenka Sikorová, Lenka Havelková, Kateřina Schwarzerová

**Affiliations:** Department of Experimental Plant Biology, Faculty of Science, Charles University, Prague, Czech Republic

**Keywords:** Actin, Plant cytoskeleton, Cell biology, Plant sciences

## Abstract

Protein complex Arp2/3 has a conserved role in the nucleation of branched actin filaments. It is constituted of seven subunits, including actin-like subunits ARP2 and ARP3 plus five other subunits called Arp2/3 Complex Component 1 to 5, which are not related to actin. Knock-out plant mutants lacking individual plant ARP2/3 subunits have a typical phenotype of distorted trichomes, altered pavement cells shape and defects in cell adhesion. While knock-out mutant Arabidopsis plants for most ARP2/3 subunits have been characterized before, Arabidopsis plant mutants missing ARPC1 and ARPC3 subunits have not yet been described. Using CRISPR/Cas9, we generated knock-out mutants lacking ARPC1 and ARPC3 subunits. We confirmed that the loss of ARPC1 subunits results in the typical ARP2/3 mutant phenotype. However, the mutants lacking ARPC3 subunits resulted in plants with surprisingly different phenotypes. Our results suggest that plant ARP2/3 complex function in trichome shaping does not require ARPC3 subunit, while the fully assembled complex is necessary for the establishment of correct cell adhesion in the epidermis.

## Introduction

The Arp2/3 complex is one of the most conserved actin nucleators, first identified in *Acanthamoeba*^1^. It is present in all eukaryotic organisms with very few exceptions^2^. It is composed of two actin-like subunits (ARP2 and ARP3) and five unique subunits (ARP2/3 complex component ARPC1 to ARPC5). With an unique mechanism of actin nucleation, the complex binds to an existing actin filament (the mother filament) and it utilizes the actin-like subunits ARP2 and ARP3 as nucleation sites initiating the polymerization of a new actin filament (the daughter filament) growing from the mother one at a characteristic 70 degrees angle^3,4^. This process is the base for the building of the branched actin network crucial for cell motility and many other cellular processes^5^.

In organisms, where subunits are coded by multiple genes, the existence of several types of isocomplexes composed of various subunit isoforms is possible^6^. It generally seems that the functional complex needs to be constituted of all its seven subunits. Protein purification analysis showed that all the subunits were present at the same stoichiometry concentration in human cells^7^ and budding yeast *Saccharomyces cerevisiae*^8^. This suggests that each subunit is necessary for the correct functioning of the complex. There are rare exceptions to this rule. These exceptions include several organisms with minimal actin cytoskeleton, where the Arp2/3 is either not coded^9^ or is modified^10^. It is not clear how the Arp2/3 complex operates here. Recently, the presence of “hybrid” complexes consisting of selected Arp2/3 subunits and vinculin was reported in the focal adhesions of chicken smooth muscle cells^11^. Here, vinculin and ARPC1 subunit can replace each other in the binding site to the Arp2/3 core. This represents the first evidence that partially assembled alternative Arp2/3 complexes that do not consist of the seven subunits may function for special cellular functions in organisms which otherwise encode all classical complex subunits.

Due to its main role in actin organization at the membrane-cytosol interphase, the lack of any of the Arp2/3 subunits is lethal in animals and severely impairing or lethal in yeast. In animals, the lack of Arp2/3 complex impairs plasma membrane remodeling and thus blocks cell migration during embryogenesis^12,13^. In yeast, the Arp2/3 complex is essential for the assembly of cortical actin patches required for endocytosis^14^ and for maintaining cellular polarity^8^.

In plants, the complex is well preserved but the absence of any of its subunits is not lethal. Nevertheless, plants lacking the functional ARP2/3 complex exhibit a number of phenotypes that suggest a conserved role for the complex in actin organization. ARP2/3 *Arabidopsis thaliana* and *Lotus japonicus* mutants known so far are characterized by irregularly shaped (distorted) leaf trichomes, which is their most evident morphological defect^15–20^. This trait was used for the identification of the eight genes of the *DISTORTED* mutant class^21^. Most genes belonging to this family were recognized as coding for ARP2/3 subunits or the SCAR/WAVE complex regulatory proteins. These include genes *arp*2, *arp3*, *arpc2* and *arpc5*^15,16,18,22^ or genes for proteins involved in the activation of the complex: *nap1* and *sra1*^22,23^. Similar T-DNA insertion lines allelic to the original *DISTORTED* mutants were also subsequently characterized^24^. Knock-out T-DNA insertional mutant lines were later characterized for the ARPC4 subunit^20,25^, also presenting the typical distorted trichome phenotype.

It appears that distorted trichomes are a hallmark mutation phenotype for plants with non-functional ARP2/3 complex or its activator complex SCAR/WAVE, and that the presence of all subunits is required for the correct trichome shaping in plants. Indeed, it has been demonstrated that ARP2/3-generated meshwork organizes the actin bundle network to enable efficient delivery of new cell wall material throughout the cell surface to maintain cell wall thickness gradients in the growing trichome branch^26,27^. Localized organization of actin mesh by activated ARP2/3 is well consistent with a conserved role of ARP2/3 known from non-plant organisms. Other defects include problems in coordinated cell expansion such as pavement cell reduced lobing in cotyledons, the presence of gaps between epidermal cells in various tissues due to cell adhesion defects, and shorter etiolated hypocotyls^15,16,19,20,22–24,28,29^. The mechanisms of ARP2/3 actin in pavement cell shaping is not fully clarified and the defect of endomembrane dynamics resulting in a different quality of deposited cell wall is one of the possible causes. Indeed, on the cellular level, defects in actin cytoskeleton-mediated transport^26,27^ and endomembrane system^30–32^ were reported in Arabidopsis. In moss *Physcomitrella patens*, dramatic phenotypes connected with defects of tip-growing cells^33^ occur in conditions where the complex is dysfunctional. ARPC1 RNAi *Physcomitrella* line results in the most severe phenotype lacking caulonemal cells and leafy phenotype^34^. Mutations in *ARP3*, *ARPC4* and *BRICK1* genes has a general phenotype of inhibited cell expansion by tip growth^35–37^. Together with the fact that both ARP2/3 and SCAR/WAVE complex subunits are localized to the tip of growing cells^36,37^ and that cortical actin is not formed during localized growth in mutants^35^, we can infer that ARP2/3-dependent actin is involved in polar growth of moss tip growing cells, probably through control of endomembrane trafficking in the growing region of cells. In seed plants, polar growth occurs in pollen tubes and root hairs. While the pollen tube is not known to have a significant phenotype in ARP2/3 mutants^38^, root hair shape^16,24^ and root hair position in trichoblasts^39^ is slightly influenced. ARP2/3 complex-driven actin polymerization is necessary during root hair initiation, as it has been localized in emerging root hairs^39–41^ and the complex is destabilized during the transition from initiation to elongation phase^39^. Thus, the ARP2/3 complex plays a role during root hair initiation, but not elongation process. An important role of ARP2/3-organized actin in membrane remodeling has been revealed during rhizobia infection of plants in a symbiotic process. The symbiotic event includes the formation of a root cell plasma membrane-derived infection thread, which allows the symbiotic bacteria to penetrate root cells^19^. Plants with incomplete or inactivated ARP2/3 complex show defects in infection thread formation and frequently develop uncolonized nodules in *Lotus japonicus*^19,42,43^ and *Medicago truncatula*^44,45^. These examples further strengthen the conserved role of ARP2/3-driven actin formation at the membrane interphase in plants. Arabidopsis mutants lacking PIR1^46^ and ARPC2^47^ proteins were further shown to be impaired in stomatal movements. The defect has been associated with the changes in actin filaments organization. Future research will determine the mechanism by which ARP2/3-controlled actin is involved in stomatal movement. To complete the list of plant processes associated with non-functional ARP2/3, we mention plant immune reaction. Tomato with silenced *ShARPC3* and wheat with knocked down *TaARPC3* have enhanced susceptibility to the powdery mildew pathogen *Oidium neolycopersici*^48^ and stripe rust fungus^49^ respectively. Arabidopsis plants lacking ARPC4 were demonstrated to be more susceptible to *Sclerotinia sclerotiorum*^50^. Also here, the mechanism associated with the role of ARP2/3-controlled actin is to be identified.

In this work, we employed the CRISPR/Cas9 methodology to generate both *arpc1* and *arpc3* Arabidopsis knockout lines. We show here that ARPC1 presence is required for the correct morphology of trichomes and cotyledon pavement cells, in accordance with previously reported ARP2/3 mutants. However, the absence of the ARPC3 subunit has no influence on either cell type, indicating that not all plant-specific ARP2/3 functions depend equally on each subunit.

## Material and methods

### Plant material

Col-0 (WT), SALK_013909.27.65 (*arpc4)*, SALK_123936.4 (*arpc5*), SALK_099449, SAIL_1210_A03C1 and SAIL_131_F01 (*arpc3*) T-DNA insertion mutant lines were obtained from The Nottingham Arabidopsis Stock Centre (NASC). The *arpc4* (SALK_013909.27.65), *arpc5* (SALK_123936.4) and *arpc2* lines were already characterized in previous research produced in our laboratory^25,29^ as were the complemented lines GFP:ARPC2 and ARPC5:mCherry^32^. Plants were grown under a 16 h-light/8 h-dark cycle (light intensity 110 mmol/m^2^/s) at 22 °C either on peat pellets or in vitro. For in vitro cultures, seeds were briefly washed with 96% ethanol and then surface-sterilized by bleach solution (sodium hypochlorite solution at the final concentration of 2.5%) for ten minutes, they were sown on half-strength Murashige and Skoog (MS) medium (1% [w/v] sucrose, 2.2 g/l MS salts [Sigma/Aldrich], 0.8% agarose, [pH 5.7]).

### Generation and identification of *A. thaliana* mutant lines by CRISPR technology

CRISPR mutant lines *arpc1a/b-c1* and *arpc3-c1* were generated using the 35S promoter-controlled CRISPR/Cas9 technology, employing the pHSE401E vector^51^. To increase the chance of a mutation, two target single-guide RNAs (sgRNAs) were designed for each gene (see Supplementary Table 1). Target gRNA sequences were identified and reverse and forward primers for them were designed with the help of the CRISPR-P web tool^52^ at http://cbi.hzau.edu.cn/cgi-bin/CRISPR. The primers used are described in Supplementary Table 1. Fragments were amplified from pCBC-DT1T2 using Q5 polymerase (New England Biolabs) according to the manufacturer’s instructions and cloned into the vector pHSE401E using the Golden Gate cloning reaction with enzymes from New England Biolabs. Col-0 plants were transformed with the resulting plasmid using *A. tumefaciens* (GV 3101 strain)-mediated floral dip^53^. After selection on hygromycin-containing media without sucrose, homozygote mutant lines were identified by sequencing PCR products containing the target sequences. Homozygotes lacking the Cas9 fragment identified by PCR were used in our experiments.

### Genotyping of *A. thaliana* T-DNA insertion mutants

SAIL and SALK *A. thaliana* mutant lines were genotyped using Lb1 and LB3 primers respectively in combination with gene-specific primers as suggested by the SALK institute website http://signal.salk.edu/tdnaprimers.2.html. Genotyping primers are listed in Supplementary Table 1.

### *A. thaliana* transgenic complementation

The complete cDNA of the *AtARPC1A* and of the *AtARPC3* genes were each cloned into 0029-pGreen expression vector containing the 35S promoter, the GFP coding gene and the terminator. The engineered vectors containing the *ARPC1A* or the *ARPC3* coding sequence fused at the N-terminal end with GFP were transformed into *A. tumefaciens* GV 3101 strain containing the pSoup helper vector. *arpc1a/b-c1* CRISPR/Cas9 mutant plants were transformed via the *A. tumefaciens*-mediated floral dip method. After selection on kanamycin, complemented plants were checked for green fluorescence and visually for the presence of wild-type trichomes. Successively, the presence of the *ARPC1A* wild-type recombinant sequence fused with GFP was confirmed by PCR and subsequent sequencing. The same protocol was followed transforming *arpc3-c1* CRISPR/Cas9 mutant plants with *A. tumefaciens* containing 35S::GFP:ARPC3 expressing vector, except for the visual check of the presence of wild-like trichomes since *arpc3-c1* mutants do not show any trichome defects. Successively we crossed the complemented *arpc3-c1* line with 35S::ARPC5:mCherry expressing line^32^. A double marker line was selected optically and *arpc3-c1* homozygotes were selected using PCR.

### Yeast mutant complementation assay

*Saccharomyces cerevisiae* temperature-sensitive strain Y06714 was obtained from the Euroscarf collection (Oberusel, Germany). The complete cDNA of *AtARPC3* and CRISPR/Cas9 mutated *AtARPC3* were cloned into the pVTU120-U vector^54^. Y06714 was transformed with the empty vector, the vector containing the WT *AtARPC3* and the vector containing the mutated *AtARPC3*. Transformed yeast cells were grown on selective SD medium lacking uracil^55^. Approximately 1 mm^2^ of positive colonies from each transformation was resuspended in 200 μL of sterile water. For each of the three resuspensions, four serial dilutions were made and 10 μL of each dilution was spotted on non-selective YAPD medium plates (1% yeast extract, 2% peptone, 2% glucose, 2% agar, 0.004% Adenine Sulfate), and let grown either at 28 °C or at 37 °C (selective temperature).

### Quantitative real-time PCR analysis

Total RNA was obtained from 5 days after germination (DAG) plants (NucleoSpin® RNA Plant Kit; #740,949, MACHEREY–NAGEL GmbH & Co., KG, Düren, Germany). Further processing (cDNA synthesis and qPCR) was done according to the protocol used by García-González et al.^31^. Samples were measured in triplicates and for three biological replicates, using premixes and raw RNA samples as negative controls. Amplification efficiencies were calculated using the LinRegPCR software^56^. Relative expression of the target gene was calculated as described by Ganger et al.^56^. The primers used are described in Supplementary Table 1.

### Trichome shape analysis

First true leaves of 14 DAG in vitro grown plants were cleared for 3 days in a solution prepared by mixing 120 g chloral hydrate, 7.5 ml glycerol and 150 ml of water, embedded in the same solution and imaged using the transmission light microscope Olympus Provis AX70. Trichome branch number was counted under transmission light microscope Olympus Provis AX70 with a 10 × objective, and branch length was measured after manual tracing on photos using the length measurement tool of ImageJ. All measurements were done in three biological replicates (at least 170 analyzed trichomes per genotype from one true leaf of at least 20 plants). Photos of cleared trichomes were captured using polarized light microscopy on Olympus AX70 Provis with a 10 × objective.

### Pavement cells analysis

*Arabidopsis thaliana* seedlings grown in vitro for 14 days were incubated for 10–20 min in an aqueous solution of propidium iodide (PI, final concentration 0.01 mg/ml) and photographed as described in^25^. Pavement cell shape analysis was quantified from skeletonized pictures in the image analysis platform Fiji^57^. Three biological replicates were carried out.

### Hypocotyl growth assay

Etiolated hypocotyl length was measured in 6 DAG etiolated plants grown *in vitro*. Pictures of seedlings were taken using a Nikon D 3200 photo camera and measured by ImageJ software. To obtain better visualization of hypocotyl pavement cells adhesion problems, plants were placed for 15 minutes in 0.1% Ruthenium Red dye water solution and washed in distilled water. Hypocotyls were immediately observed under stereomicroscope Olympus SZX7 equipped with an Olympus DFPL2X-4 objective lens (NA 0.2) connected to a digital camera Canon EOS 760. Images were analyzed using the Fiji platform. The level of staining with Ruthenium Red was evaluated in pictures that were processed. Briefly, weighted conversion of RGB pictures to 8-bit was carried out to enhance the signal intensity of the dye. Then, ROIs of approximately 1 mm^2^ were selected manually to enclose a fragment of the hypocotyl and dye staining intensity was measured as an integrated density value in the 8-bit processed pictures. Approximately 40 hypocotyls for each variant were analyzed in three biological replicates.

### Isolation of proteins and co-immunoprecipitation

For total protein fraction isolation, approximately 1 g of 10 DAG Arabidopsis seedlings were frozen in liquid nitrogen and homogenized with mortar and pestle. The frozen powder was mixed 1:1 (v/w) with 2 × concentrated MES buffer (25 mM MES; 5 mM EGTA; 5 mM MgCl_2_; 1 M glycerol; pH = 6.9) supplemented with a protease inhibitors cocktail (Sigma-Aldrich, P9599) and was let thaw on ice. The sample was then centrifuged at 3000 g for 15 min at 4 °C and the supernatant was centrifuged at 25,000 g for 30 min at 4 °C. The supernatant contained 2.5 mg proteins/1 ml (determined using Bio-Rad Protein Assay). This protein fraction was used for co-immunoprecipitation (input). 1 ml of protein extract was mixed with 50 µl of magnetic beads from µMACS™ GFP Isolation Kit (Miltenyi Biotec) and the mixture was incubated on ice while mildly shaking for 30 min. The extract was then loaded into the column in a magnetic stand and GFP-tagged proteins and their interactors were isolated according to the manufacturer protocol. Isolated immunoprecipitated proteins (the whole amount of eluted fraction, 50 µl) as well as respective inputs (20 µl of extracts corresponding to 50 µg of total proteins) were separated using SDS PAGE electrophoresis and transferred onto nitrocellulose membrane by electro-blotting (SemiDry, BioRad). Western blots were probed with rabbit anti-GFP (1:5000; Agrisera AS152987) and rabbit anti-RFP (1:6000; Abcam, ab167453). Full blots are supplied in Fig. S6.

### Reporter line generation and GUS staining

For the generation of the *pARPC1::GUS* reporter line, a region of 1794 bp (between positions − 42 to − 1791 upstream of the *ARPC1a* gene starting codon) was amplified and cloned into the binary vector pRD410^58^. T2 generation of stably expressing plants obtained using *A. tumefaciens* (GV 3101 strain)-mediated floral dip and selection on kanamycin was used for further experiments. For GUS staining, 7 DAG in vitro grown plants were harvested, and histochemical staining was performed according to the protocol described in^31^.

### Transient transformation of *N. benthamiana*

*Nicotiana benthamiana* leaves were transiently transformed using the agroinfiltration method. *Agrobacterium* was cultivated overnight in a YEB medium with corresponding antibiotics. The culture was centrifuged at 3600 g for 10 min and washed three times in infiltration buffer (10 mM MgCl_2_, 10 mM MES, pH = 6.5) and finally resuspended to OD_600_ = 0.6 in an infiltration solution with 200 µM acetosyringone. The bacterial solution was then infiltrated into the leaf mesophyll using a 2 ml syringe. Infiltrated *N. benthamiana* plants were then cultivated for three days before microscopic observation.

### Confocal microscopy

Confocal microscopy of GFP tagged ARP2/3 subunits in *N. benthamiana* and *A. thaliana* plants was performed using confocal laser scanning microscope Leica SP8. For image acquisition, objective HC PL APO CS2 63 × with NA = 1.2 was used. The excitation was set at 488 nm and emission to 490–550 nm for GFP, and excitation was set to 561 nm and emission to 580–650 nm for mCherry. The pinhole was set to 1AU.

### Statistical analysis

Statistical analysis was conducted using R software. Data were fitted with a linear model (LM) or linear mixed-effects model (LMM), with the fixed effect being the genotype and the random factor being the number of biological replicates (for LMM). Log- or squared-root transformations were used to achieve normality of residuals when required (as specified in figure legends). Analysis was followed by posthoc Tukey multiple comparison analysis.

## Results

### Characterizations of CRISPR/Cas9 mutated plants

In *A. thaliana,* the *ARPC1* gene is duplicated and two very similar copies are located on chromosome 2 in relatively close proximity: *ARPC1A* (AT2G30910) and *ARPC1B* (AT2G31300)^24^. T-DNA insertion lines for each of both copies of the *ARPC1* gene do exist, but they have no typical ARP2/3 mutant phenotype, suggesting that the two *ARPC1* genes are functionally redundant. The double mutant line is not available due to the reduced distance between the two genes.

**Figure 1:**
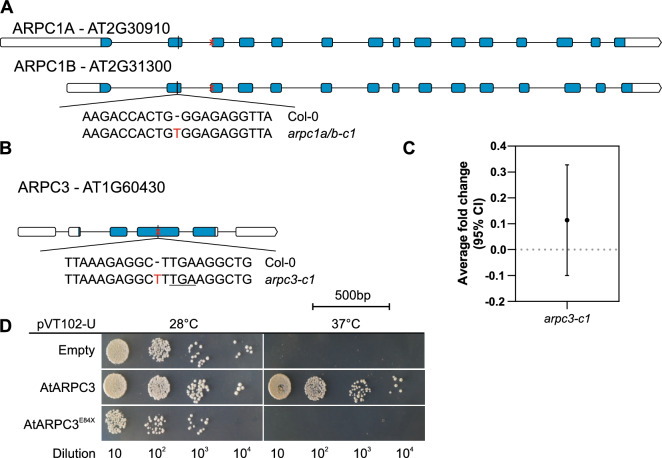
Characterization of the newly generated *Arabidopsis thaliana* CRISPR/Cas9 mutant lines *arpc1a/b-c1* and *arpc3-c1*. Schematic representation showing the position of the single-nucleotide insertions leading to frameshift mutations and early transcript termination for both (**A**) *ARPC1A/ARPC1B* and (**B**) *ARPC3* genes. (**C**) Further molecular characterization of the *arpc3-c1* line via qRT-PCR with specific primers for the *ARPC3* coding sequence shows negligible gene expression compared to wild-type plants. (**D**) Yeast complementation assay was used to assess the functionality of both full-length and truncated versions of ARPC3 at selective temperature (37 °C). While full-length ARPC3 can recover the growth phenotype of *Δarc18* yeast strains, truncated ARPC3 fails to reinstate its viability. Serial yeast culture densities were used.

To generate *arpc1* and *arpc3* knock-out mutants, we used CRISPR/Cas9 technology. We were able to generate several different insertion/deletion mutants both for *ARPC1A/B* and *ARPC3*. For the generation of *arpc1a/b*, we selected a homozygous plant carrying the same single nucleotide insertion in both genes, leading to a frameshift, and resulting in a premature stop codon in both genes (*arpc1a/b-c1*, Fig. 1A). For *arpc3*, we were able to create several mutant lines, from which we selected and used in our study one line containing one single T nucleotide insertion creating a stop codon approximately in the middle of the coding region (*arpc3-c1*, Fig. 1B).

**Figure 2:**
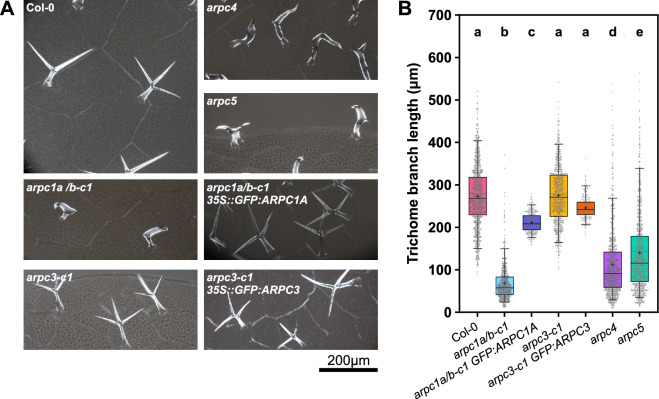
*arpc1a/b-c1* but not *arpc3-c1* show a distorted phenotype. Trichome evaluation of first true leaves from 14DAG plants reveals the “distorted”-like phenotype in *arpc1a/b-c1*, but not in *arpc3-c1* knockout lines. (**A**) Representative polarized light microscopy images of Col-0, *arpc1a/b-c1*, *arpc1a/b-c1 35S::GFP:ARPC1*, *arpc3-c1*, *arpc3-c1 35S::GFP:ARPC3*, *arpc4*, *arpc5* trichomes. (**B**) Quantification of trichome branch length. Statistical analysis was performed by fitting a linear mixed model on double squared root-transformed data followed by posthoc Tukey contrasts. Different letters denote statistical significance (*p* < 0.01). n = 170–970 trichome cells.

Homozygous plants *arpc1a/b-c1* showed a typical distorted phenotype (see hereinafter). Interestingly, *arpc3-c1* mutant generated by CRISPR/Cas9 showed no macroscopic phenotype and mutant plants had WT-like trichomes, despite the fact that in WT, the gene is expressed in this cell type (Figs. 2A, S1A). Our previous study on the promoter activity of ARP2/3 subunits showed that *ARPC3* appears to be expressed in all cell types and tissues like the other subunits, including cotyledon and true leaves^31^. Here we analyzed the promoter activity *ARPC1A* gene native tissue-specific expression pattern through the histochemical staining of GUS activity in plants expressing the *pARPC1A::GUS* construct. We found that the gene for this subunit is also expressed in the whole plant with a pattern similar, but not identical to the other subunits' pattern (Fig. S1B). Promoter activity was detected around vascular bundles, shoot and root apical meristems, corresponding to the already observed patterns. However, small differences in expression distribution include clear expression in the stele up to the quiescent center, while this was not the case for the other studied subunits in^31^. Also, while subunits are generally found in columella cells, *ARPC1A* promoter activity in that region was reduced. We next tested the expression level of *ARPC3* gene in the CRISPR mutated line. Quantitative real-time PCR showed that mRNA of the mutated *ARPC3* gene is expressed at a negligible level (Fig. 1C). We further tested if the possibly expressed truncated protein could be still functional. We performed a yeast complementation assay on a *Saccharomyces cerevisiae* strain *Δarc18* containing a mutated gene coding for a thermosensitive isoform of the yeast *ARPC3/ARC18* protein. We took advantage of previously shown experiments demonstrating that plant ARPC3 can rescue the yeast mutant phenotype^49^. We transformed the mutant yeast strain with a plasmid containing either the wild type, the mutated *ARPC3* gene, or an empty plasmid. As shown in Fig. 1D, all types of transformed yeast were able to grow at the non-restrictive temperature of 28 °C while just yeast transformed with wild type ARPC3 were able to do so at the restrictive temperature of 37 °C. This result confirmed that the truncated ARPC3 protein is not functional.

To confirm that plants without functional ARPC3 do not show the typical distorted phenotype, we searched for independent knock-out mutants in Arabidopsis mutant databases. We tested the SALK_099449 line that has been characterized as a knock-out by Sun and colleagues^48^. However, our results indicated that SALK_099449 is in fact an overexpression line (Fig. S2A,B). We also tested SAIL_1210_A03_C1 and SAIL_131_F01, which contain a T-DNA insertion at the beginning of the transcribed region of the *ARPC3* gene (Fig. S2A). The SAIL_1210_A03_C1 line was confirmed as a true *arpc3* knock-out mutant, but SAIL_131_F01 was found to be an overexpressing line (Fig. S2B). It is important to note that the SAIL_1210_A03_C1 line is described as a double mutant containing a further T-DNA insertion on a different chromosome in the SALK database; however, our repeated attempts to outcross this second mutation made clear that more unmapped T-DNA insertions are present in other loci in the genome, which made impossible to obtain a single T-DNA insertional mutant homozygous line for *ARPC3*. Nevertheless, also this mutant line showed WT-like trichomes without morphological abnormalities (Fig. S3).

### Plant morphological analysis

Distorted trichomes phenotype is reported as the most typical phenotypic representation of an inactive ARP2/3 complex. Therefore, *arpc4* (SALK_013909.27.65) and *arpc5* (SALK_123936.4) mutant lines that have been used in our previous studies^25,29^ were used as positive controls in this present study. Knockout *arpc1a/b-c1* plants showed the typical characteristics of the *DISTORTED* class mutants (Fig. 2A, see *arpc4* and *arpc5* mutant), with distinctly short and malformed trichome branches. However, none of the homozygote mutant *arpc3* plants had any distorted trichomes or differed macroscopically from wild-type Col-0 plants (Fig. 2A). Quantitative analysis of trichome shape confirmed typically short trichome branches in *arpc4*, *arpc5* and *arpc1a/b-c1* mutant plants (Fig. 2B). The only distinctive trait of *arpc1a/b-c1* plants was a lower number of trichome branches when compared to other tested mutants (Fig. S4). *arpc3-c1* mutants’ trichomes shape and size were comparable to wild type (Fig. 2).

In order to test for specific defects of mutant lines, we constructed expression plasmids with *GFP: ARPC1A* and *GFP:ARPC3* genes driven by the 35S promoter, expressed them in respective mutant plants and analyzed if they rescued the phenotype. GFP: ARPC1A protein had cytoplasmic localization (Fig. S5A,B,C); its expression rescued distorted trichome phenotypes when expressed in the *arpc1a/b-c1* line (Fig. 2A,B). *GFP:ARPC3* was expressed in transformed plants and the protein had cytoplasmic localization (Fig. S5D,E) and occasionally co-localized with peroxisomes (Fig. S5E). The *arpc3-c1* line expressing 35S:: GFP:ARPC3 had WT-like trichomes (Fig. 2B).

**Figure 3:**
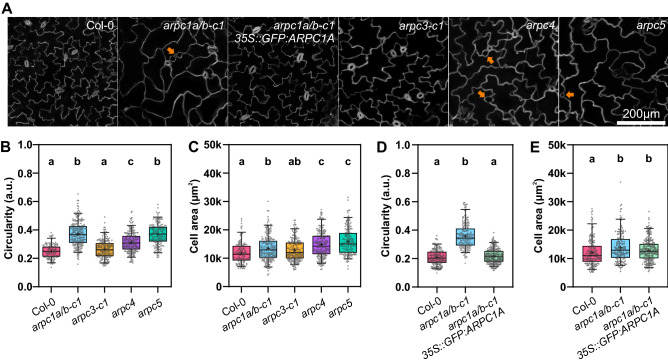
Pavement cell analysis of 14-day-old seedling cotyledons. (**A**) Exemplary images of the maximum projection of z-stacks around pavement cells' middle plane. Orange arrows indicate gaps between cells. (**B**,**C**) Quantification of epidermal cell circularity and area for Col-0 and mutants lacking single ARP2/3 complex subunits (*arpc1a/b-c1*, *arpc3-c1*, *arpc4*, *arpc5*). Statistical analysis was performed fitting a linear mixed model on squared root-transformed data followed by a posthoc Tukey HSD multiple comparison test. n = 137–226 cells. Different letters denote statistical significance (*p* < 0.05). (**D**,**E**) Quantification of epidermal cell circularity and area for Col-0, *arpc1a/b-c1,* and its corresponding rescue line. Statistical analysis was performed fitting a linear mixed model on either log- or squared root-transformed data (circularity and area, respectively) followed by posthoc Tukey contrasts. Different letters denote statistical significance (*p* < 0.05). n = 191–263 cells.

Another hallmark phenotype of the *DISTORTED* group of mutants is the reduced shape complexity of cotyledon pavement cells. We tested our CRISPR-generated mutants for pavement cell shape and size. Quantitative morphological analysis of cotyledon pavement cells confirmed that *arpc1a/b-c1* mutants have less lobed pavement cells, comparable to *arpc4* and *arpc5* (Fig. 3A,B). We observed that gaps were present between *arpc1a/b-c1* pavement cells, another trait of the distorted phenotype (arrows in Fig. 3A). Similar to the trichome phenotype, *arpc3-c1* cotyledon pavement cells morphology was not distinguishable from wild type pavement cells, and no gaps were present between the cells (Fig. 3A,B). The analysis confirmed increased cell area in all mutants tested (Fig. 3C). In a separate experiment, we tested the *arpc1a/b-c1* line expressing GFP:ARPC1A for cell shape and size rescue. GFP:ARPC1A expression rescued cell shape defects of the mutant line (Fig. 3D), but the phenotype of increased cell area was just partially rescued (Fig. 3E), probably due to either overexpression, or the impact of the GFP tag on the complex functional efficiency.

**Figure 4:**
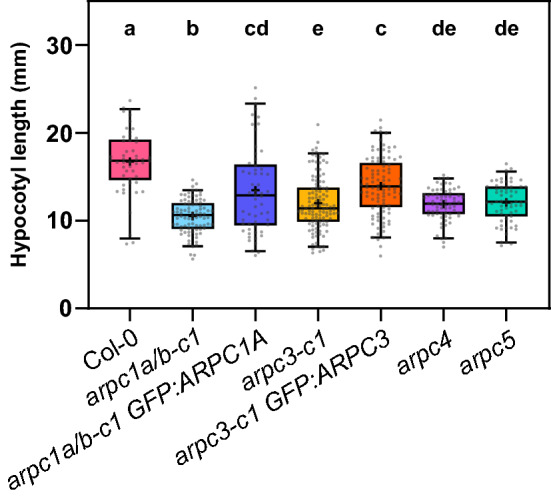
Dark-grown hypocotyl length evaluation. Hypocotyl length analysis of etiolated seedlings for the lines generated in this study (*arpc1a/b-c1*, *arpc3-c1*), their respective fluorescent-labeled rescue lines (*arpc1a/b-c1 35S::GFP:ARPC1A*, *arpc3-c1 35S:GFP:ARPC3*) and control lines (Col-0, *arpc4*, *arpc5*). Hypocotyl analysis of dark-grown 6-day-old plants shows reduced hypocotyl length for all lines lacking single ARP2/3 complex subunits, and a partial recovery of the corresponding rescue lines. Statistical analysis was performed fitting a linear mixed model on the original data followed by a posthoc Tukey HSD multiple comparison test. n = 50–117 plants. Different letters denote statistical significance (*p* < 0.05).

**Figure 5:**
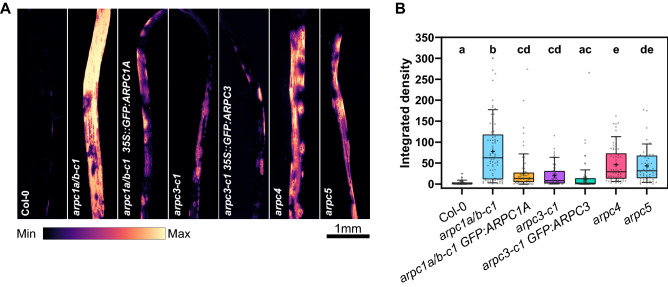
Cell adhesion analysis of etiolated hypocotyls. Increased cell adhesion problems are observed in plants with altered ARP2/3 complex subunit stoichiometry, as revealed by the staining of de-esterified pectins by Ruthenium red in 6-day-old etiolated seedlings. (**A**) Representative micrographs (mpl-magma LUT) of dark-grown plants stained for 15 min in a 0.1% Ruthenium red solution. (**B**) Quantification of the extent of dye penetration as integrated density in the labeled hypocotyls. Statistical analysis was performed fitting a linear mixed model on the original data followed by posthoc Tukey contrasts. Different letters denote statistical significance (*p* < 0.01). n = 53–67 plants.

Single subunit ARP2/3 mutant Arabidopsis plants were previously shown to have shorter etiolated hypocotyls^15–17,20^. Therefore, we tested CRISPR/Cas9-generated mutants for these defects. As shown in Fig. 4, *arpc1a/b-c1* seedlings grown in the dark displayed shorter hypocotyls comparable to *arpc4* and *arpc5* mutants phenotype. *arpc1a/b-c1* complemented with GFP:ARPC1A showed partial recovery of the WT phenotype with varying degrees of rescue among seedlings. Interestingly, also *arpc3-c1* mutants did reveal a phenotype with shorter hypocotyls and the phenotype was partially rescued in the complemented line (Fig. 4). Plants lacking functional ARP2/3 complex have been reported to have defects in pavement cell adhesion in cotyledon, true leaves and hypocotyls^15–17,20,24,29^. These defects are especially pronounced in etiolated hypocotyls. We tested whether cell adhesion defects can be detected in our new mutants produced by CRISPR/Cas9 method. For this purpose, we used the Ruthenium Red dye, which stains pectins in epidermal cells with cell adhesion defects. We show here that all tested ARP2/3 mutants including CRISPR-generated new lines had cell-adhesion defects in etiolated hypocotyls (Fig. 5A). Quantification analysis showed that *arpc1a/b-c1* mutants had the strongest defect, represented by an increased dye internalization and concentration in hypocotyl tissues, while *arpc4* and *arpc5* mutants had slightly less severe phenotype, and *arpc3-c1* had the mildest phenotype (Fig. 5B). Here, GFP:ARPC1A expression rescued significantly the defect of *arpc1a/b-c1,* and the weak *arpc3-c1* defect was rescued in mutants expressing GFP:ARPC3 (Fig. 5B). The distribution of data in *arpc1a/b-c1* expressing GFP:ARPC1A reflects the fact that the plant population was not responding homogeneously.

### Co-immunoprecipitation of ARPC3 and ARPC5

**Figure 6:**
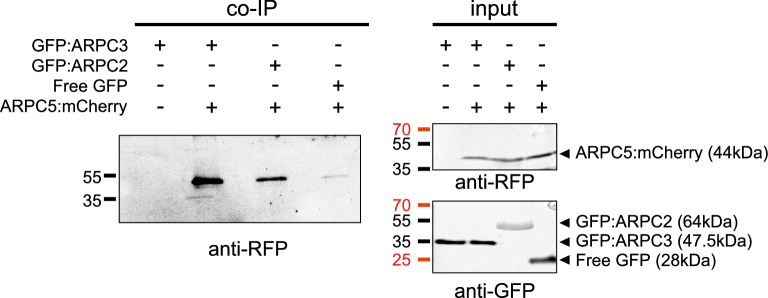
ARPC3 and ARPC5 interact in vivo*.* Co-immunoprecipitation assays prove the interactions between ARPC3 and ARPC5 using the magnetic bead GFP isolation method. Either GFP-tagged ARPC3, GFP-tagged ARPC2A (positive control), or free GFP (negative control) were co-expressed with mCherry-tagged ARPC5. Protein interactions in the immunoprecipitated samples (left panel, co-IP) were detected by immunostaining with anti-RFP antibody. Protein extracts (right panel, input) were immunostained with anti-RFP and anti-GFP. Blots were cropped; original uncropped blots are presented in Fig. S6.

We concluded that our new *arpc1a/b-c1* mutant is a true ARP2/3 mutant showing all phenotypic traits reported previously for plants lacking one of ARP2/3 subunits. However, the *arpc3-c1* mutant line generated using CRISPR/Cas9 method showed a typical ARP2/3 mutant phenotype only in the case of hypocotyl growth and hypocotyl epidermal cell adhesion defects, while such defects were not apparent between cotyledon epidermal cells. This fact challenges the assumption that only the fully assembled ARP2/3 complex is functional and necessary for all the roles it plays within the plant organism. To prove that the ARPC3 subunit is indeed interacting with other ARP2/3 subunits and is a putative component of the ARP2/3 complex in Arabidopsis, we established a line stably expressing GFP:ARPC3 and ARPC5:mCherry. Proteins isolated from this line were used in co-immunoprecipitation experiments, where magnetic beads with anti-GFP antibodies were employed to bind GFP-tagged proteins. Our experiment proved the presence of ARPC5:mCherry in the bound fraction (Fig. 6). As a positive control, we used the Arabidopsis line stably expressing both GFP:ARPC2 and ARPC5:mCherry subunits, which have been previously shown to interact and co-sediment together^32^. The line co-expressing ARPC5:mCherry and free GFP was used as a negative control. In this last experiment, we demonstrate that the ARPC3 subunit is interacting with other subunits of the ARP2/3 complex, as it co-sediments with ARPC5, similarly to the existing binding between ARPC2 and ARPC5 (Fig. 6).

## Discussion

In this work, we prepared using CRISPR/Cas9 technology two Arabidopsis mutants lacking subunits of the ARP2/3 complex that have not been described before: *arpc1a/b-c1* and *arpc3-c1*. In the case of the genes for the ARPC1 subunits, CRISPR/Cas9 was the only way to obtain a knock-out line mutant, because ARPC1 is encoded by two genes, *ARPC1A* and *ARPC1B*, which are localized very close together in the genome. Although T-DNA insertion mutants are available for each of the two genes, a double mutant cannot be constructed due to the low probability of recombination. Because neither of the single mutants exhibits a distorted phenotype (data not shown), but the *arpc1a/b-c1* double mutant does, we can infer functional redundancy of the products of the two genes in Arabidopsis. *arpc1a/b-c1* mutant line showed all the typical characteristics of the ARP2/3 mutants with severely distorted trichomes, less lobed pavement cells in cotyledons, and pronounced cell adhesion defects both in cotyledons and etiolated hypocotyls. This corresponds to the characteristics of the already described *arpc1* mutant in *Lotus japonicus*^19^ and is in concordance with moss *arpc1* mutant^34^. Our results were confirmed by the rescue of *arpc1a/b-c1* line with full-length ARPC1A fused to GFP.

To our surprise, CRISPR/Cas9 mutants with a premature stop codon in the gene encoding the ARPC3 subunit did not have the obvious phenotypic features of ARP2/3 mutants, such as distorted trichomes and epidermal cell shape problems in cotyledons. Following results suggest that the ARPC3 subunit in the *arpc3-c1* line is not functional. First, the mutant gene is expressed at a very low level relative to the WT gene. Second, functional analysis has shown that the trimmed ARPC3 is unable to complement the mutant in yeast, whereas the WT gene complements yeast loss-of-function strain, as already shown in a similar study^49^. Third, the independent T-DNA insertional SAIL_1210_A03_C1 line also did not show typical distorted trichomes, in agreement with our observations for the CRISPR/Cas9-generated *arpc3-c1*. During our search for other independent mutant lines with knocked-out *ARPC3* gene, we found out that the previously published T-DNA insertion line for the *ARPC3* gene in *Arabdiopsis*^48^ is not a knock-out mutant.

Since we were sure that we had a CRISPR/Cas9 knock-out mutant, we searched for other phenotypes of ARP2/3 complex mutants. These are defects in the hypocotyl, such as altered hypocotyl growth in the dark and problems in epidermal cell adhesion^15–17,20,24^. We show here that these phenotypic manifestations can be detected in the *arpc3-c1* mutant. Quantitative analysis proved that the defects in the hypocotyl are comparable to other ARP2/3 mutants.

Our unexpected results led us to further investigate the hypothesis of whether the ARPC3 protein is assembled to the ARP2/3 complex. We show that the GFP:ARPC3 protein co-sediments with the ARPC5:mCherry protein, as it has already been shown for ARPC2 and ARPC5 proteins^32^. These results confirm that the ARPC3 protein is part of the complex as it co-sediments with the ARPC5 subunit, which according to crystallographic studies does not directly interact with the ARPC3 protein in the Arp2/3 complex^4^.

By preparing *arpc1* and *arpc3* mutants, we currently have a full set of Arabidopsis mutants for all subunits of the ARP2/3 complex. This unique situation allows us to assess the relative importance of individual subunits for the performance of the complex. Based on our results, it is clear that the role of ARP2/3 in hypocotyl growth and the control of epidermis integrity requires a fully assembled complex, because the loss of any subunit leads to the described phenotypic manifestations^15–17,20^. An explanation of the missing cell adhesion phenotype in leaves and cotyledons is that cell adhesion defects, which were present in etiolated hypocotyls of *arpc3-c1* line, are too weak to be detected in cotyledons and leaves. We cannot exclude the possibility that under conditions allowing extensive cell growth, as occurs in etiolated hypocotyls, defects in cell adhesion would also appear in the leaves and cotyledons. Interestingly, the presence of the ARPC3 subunit is not required for the proper formation of trichomes and puzzle-like shaped epidermal cells of the cotyledons, while other ARP2/3 subunits are needed.

Mutant human and yeast cells without ARPC3 protein have the least dramatic phenotype when compared to the loss of other subunits. The loss of ARPC3 in human cells does not cause the disassembly of the complex, but it results in a formation of a complex which is very inefficient due to problems connecting to the mother actin filament^59^. This results in defects in lamellipodium formation and subsequent death at the very beginning of embryonic development^60^. Similarly, *Saccharomyces cerevisiae* knock-out mutants for the gene coding ARPC3 have a less severe phenotype compared to the knock-out mutants of other ARP2/3 subunits, suggesting that also in yeast the complex lacking ARPC3/Arpc3 still retains part of its functionality^8^. However, the lack of ARPC3 is lethal in *Schizosaccharomyces pombe* because of defects in endocytosis^61^. In the plant kingdom, the role of ARPC3 has been only little investigated. There is consistent evidence from studies on wild and domesticated tomato (*Solanum habrochaites* and *S. lycopersicum*) and on wheat (*Triticum aestivum*) that the expression of *ARPC3* is induced following fungal infection. In these plants, ARPC3 seems to be involved in the plant defense response through mechanisms that need to be elucidated^48,49^ In respect to our finding that *arpc3* Arabidopsis plants have rather mild phenotype in comparison to other complex subunits, it will be interesting to study the role of various other ARP2/3 subunits in plant immunity. Other studies indicate that the expression of *ARPC*3 is strong in pollen relative to the other subunits^24^. Interestingly, our previously published study confirmed that *ARPC3* is expressed in leaves and cotyledons^31^, plant tissues where the absence of the protein does not seem to have any obvious morphological effect. Therefore, the role of ARPC3 in pollen deserves further investigation. Although mutants lacking individual subunits of the SCAR/WAVE activator complex *nap1* and *pir1* showed that a significant proportion of their siliques are shorter with reduced seed set^62^, we found no evidence of shorter siliques of *arpc3-c1* mutants.

ARPC3 is a highly conserved protein^63^. Within the complex, ARPC2 and ARPC4 are the complex core, and the loss of these subunits results in complete complex disassembly^8^. The integrity of the complex is not dependent on the presence of ARP2 and ARP3 subunits, which play a major role in actin monomer binding^4^. ARPC5 is a less conserved subunit and its role is not yet clarified but there is evidence that it is both structural and regulative^4,8^. ARPC1 subunit has a beta-propeller structure and seems to have several roles in the complex that range from structural, connecting the complex to the mother filament, to regulatory, interacting with activators^64^. Moreover, the loss of ARPC1 results in the inability of the ARPC5 subunit to bind to the complex^65^. Knocking out ARPC1 in yeast results in the most severe phenotype with 100% lethality. Our results support ARPC1's importance in the complex, as the loss of ARPC1 in Arabidopsis causes a strongly distorted phenotype. ARPC3 subunit’s role within the ARP2/3 complex is somehow less clear. More recent studies on the complex 3-dimensional structure indicate that ARPC3’s position in the complex helps both ARP3 and ARP2 connect to the mother filament^4^. ARPC3 interacts with the SCAR subunit of the SCAR/WAVE complex^43,66,67^. SCAR has 5 isoforms in Arabidopsis^68^. SCAR1 and SCAR2 interact with ARPC3^66,67^, while SCAR3 interacts with ARP3, ARPC2 and ARPC3^67^. The role of ARPC3 in interaction with the activation complex would be consistent also with the finding that in mammals, the absence of ARPC3 results in formation of inefficient ARP2/3 complexes^59^. The existence of multiple SCAR isoforms with different interactions with the ARP2/3 complex provides a possible explanation for the weaker and tissue-specific phenotypes of *arpc3* mutants, because other subunits may mediate SCAR interaction with the complex under specific conditions.

In summary, we have created two new mutants using CRISPR/Cas9 technology that complete the list of Arabidopsis ARP2/3 subunit mutants. The loss of ARPC1 results in a typical distorted phenotype—affected trichome and cotyledon pavement cell morphogenesis and cell adhesion problems. In contrast, the loss of ARPC3 subunit causes a conditional defect in hypocotyl length and hypocotyl cell adhesion only. The expression data together with the absence of distorted trichomes phenotype in *arpc3-c1* mutants suggest that partially assembled ARP2/3 complex lacking ARPC3 is functional in control of trichome and cotyledon epidermis shaping. However, a fully assembled complex is required to maintain proper cell adhesion in elongating hypocotyl epidermal cells.

## Supplementary Information

Supplementary Information 1.

Supplementary Information 2.

Supplementary Information 3.

Supplementary Information 4.

Supplementary Information 5.

Supplementary Information 6.

Supplementary Information 7.

Supplementary Information 8.

## Data Availability

All data generated or analyzed during this study are included in this published article. The datasets from this study are published on the OSF Repository^69^ (https://doi.org/10.17605/OSF.IO/XNSQF).
