# Author Correction: CRISPR‑Cas9 Arabidopsis mutants of genes for ARPC1 and ARPC3 subunits of ARP2/3 complex reveal differential roles of complex subunits

**DOI:** 10.1038/s41598-023-32386-x

**Published:** 2023-04-12

**Authors:** Erica Bellinvia, Judith García‑González, Petra Cifrová, Jan Martinek, Lenka Sikorová, Lenka Havelková, Kateřina Schwarzerová

**Affiliations:** Department of Experimental Plant Biology, Faculty of Science, Charles University, Prague, Czech Republic

Correction to: *Scientific Reports* https://doi.org/10.1038/s41598-022-22982-8, published online 28 October 2022

The original version of this Article contained an error in the Funding section.

“The work was supported by the Ministry of Education, Youth and Sports of the Czech Republic, project No. 19-10845S.”

now reads:

“The research was supported by Czech Science Foundation, Grant No. 19-10845S.”

The original Article has been corrected.

