# Supplementary figures and images for: CRISPR-Cas9 Arabidopsis mutants of genes for ARPC1 and ARPC3 subunits of ARP2/3 complex reveal differential roles of complex subunits
Source: Sci Rep. 2022 Oct 28;12:18205. doi: 10.1038/s41598-022-22982-8 (PMC9616901; doi:10.1038/s41598-022-22982-8)

# Supplementary figure 6

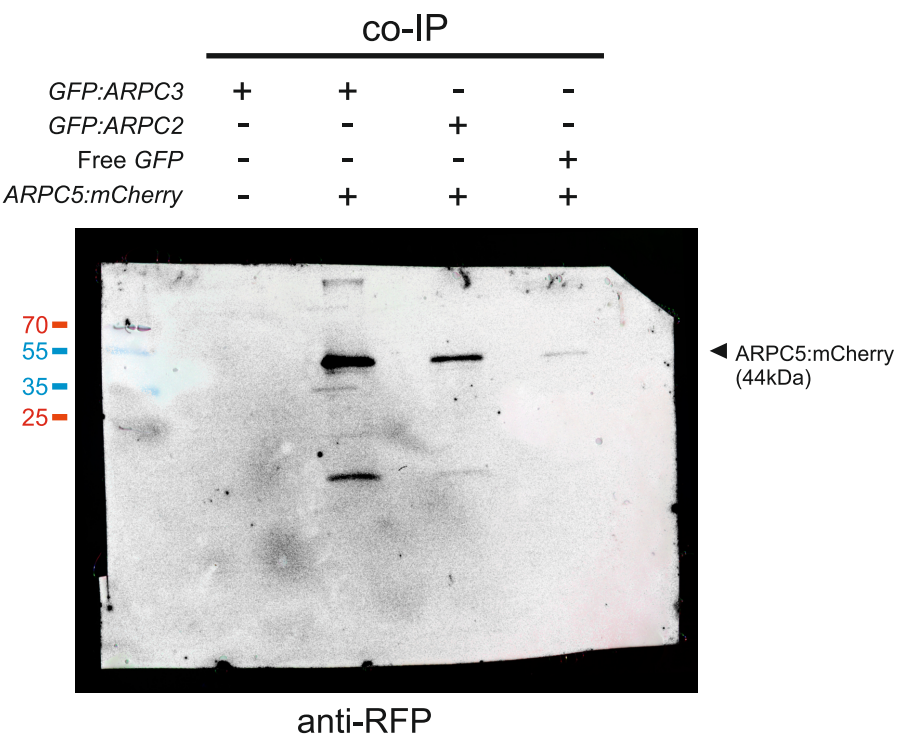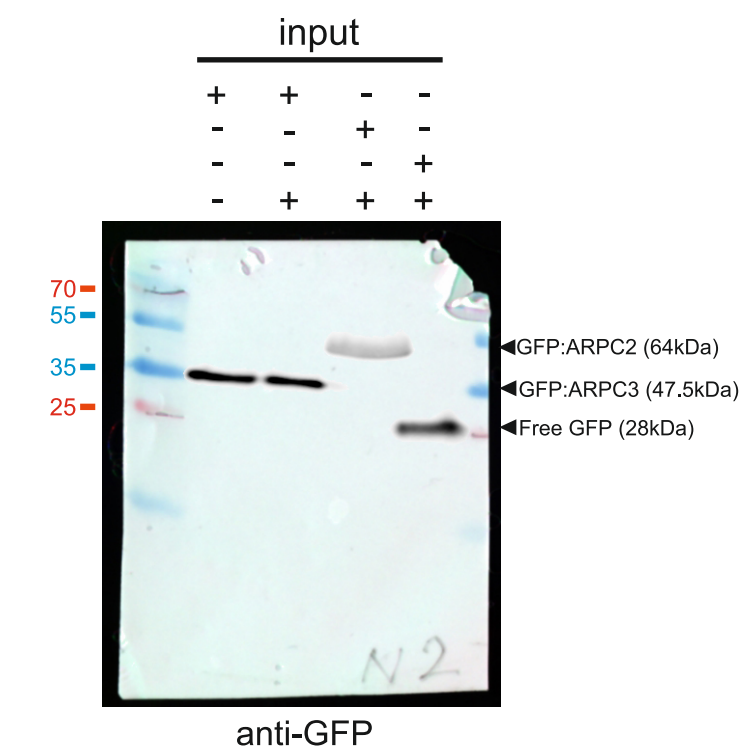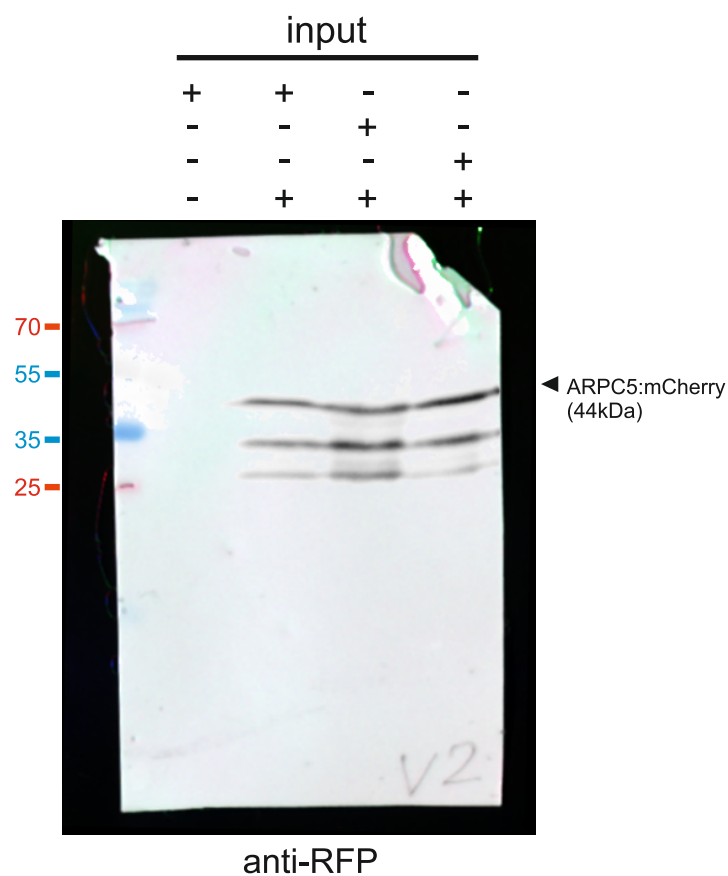

Supplement: Supplementary file 1 — Supplementary Information 1. [file 41598_2022_22982_MOESM1_ESM.pdf]

# Supplementary figure 1

**A**

*pARPC3::GUS*

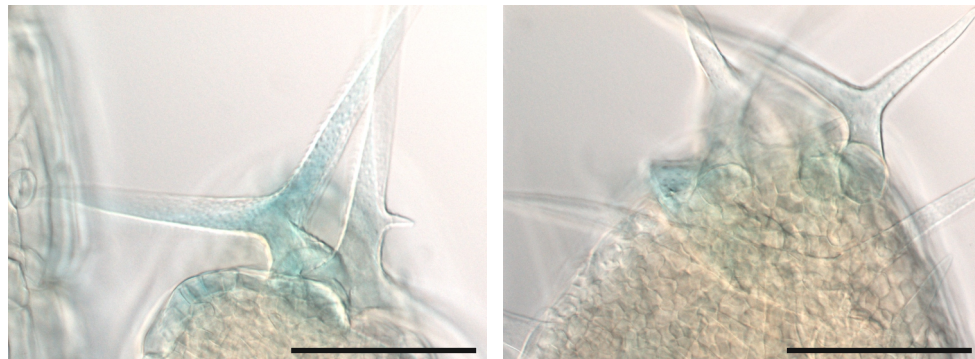

Scalebar = 100 $\mu$ m

**B**

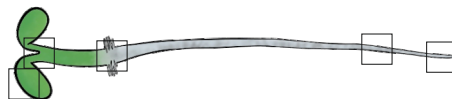

*pARPC1A::GUS*

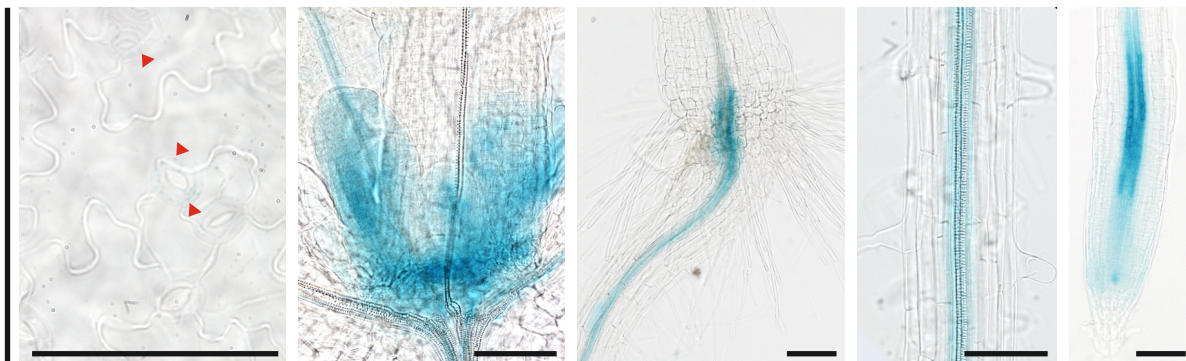

Scalebar = 200 $\mu$ m

Supplement: Supplementary file 3 — Supplementary Information 3. [file 41598_2022_22982_MOESM3_ESM.pdf]

**A**

ARPC3 - AT1G60430

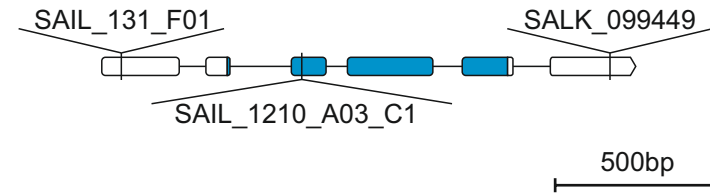**B**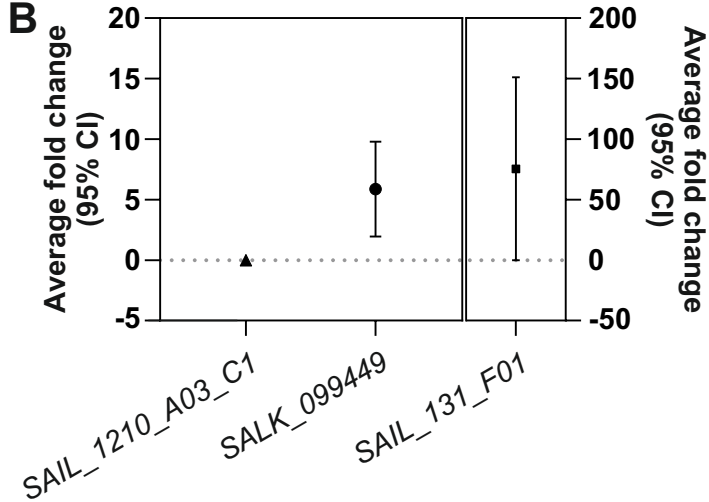

Supplement: Supplementary file 4 — Supplementary Information 4. [file 41598_2022_22982_MOESM4_ESM.pdf]

SAIL\_1210\_A03\_C1

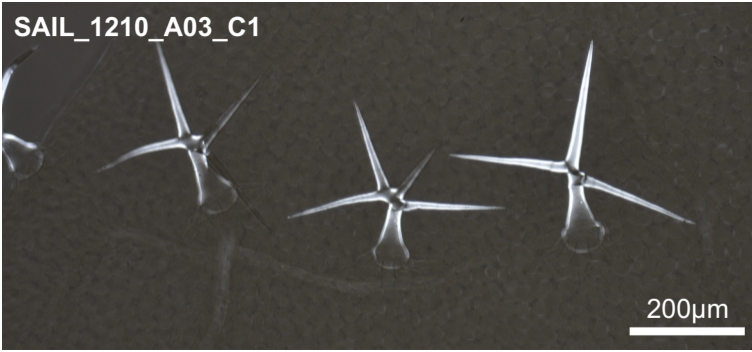

Supplement: Supplementary file 5 — Supplementary Information 5. [file 41598_2022_22982_MOESM5_ESM.pdf]

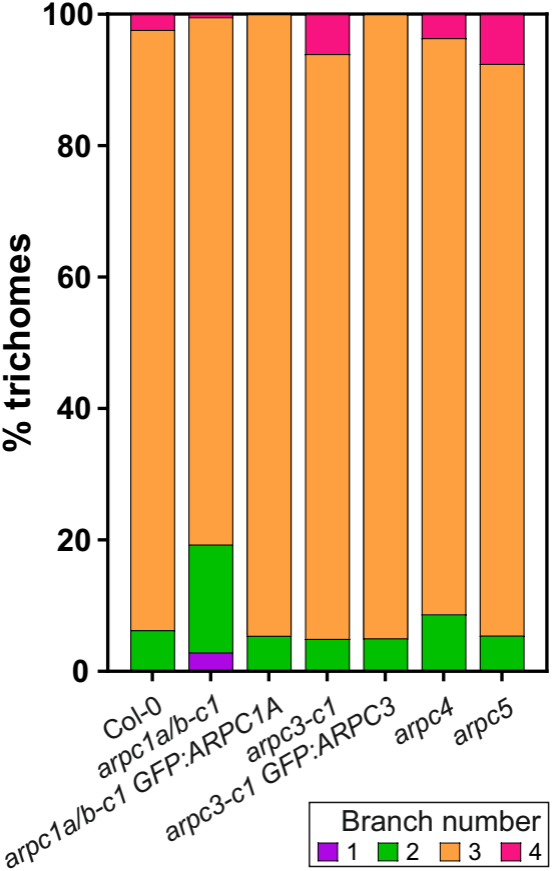

Supplement: Supplementary file 6 — Supplementary Information 6. [file 41598_2022_22982_MOESM6_ESM.pdf]

Supplementary figure 5

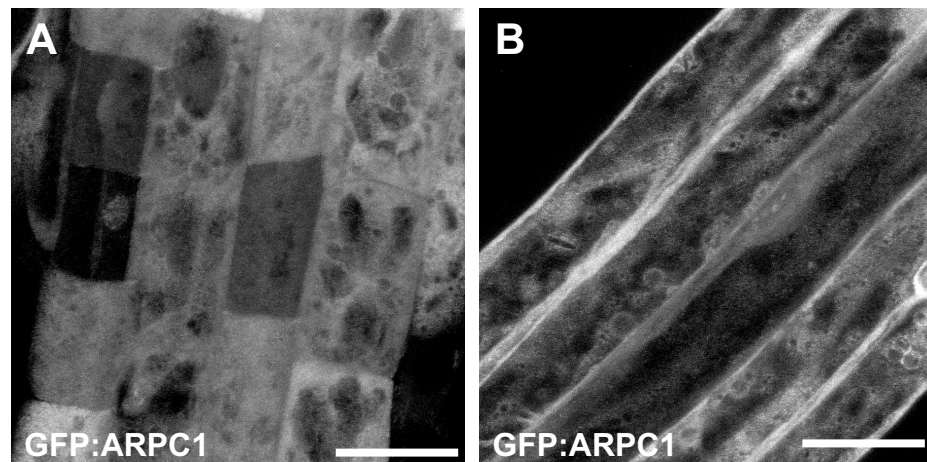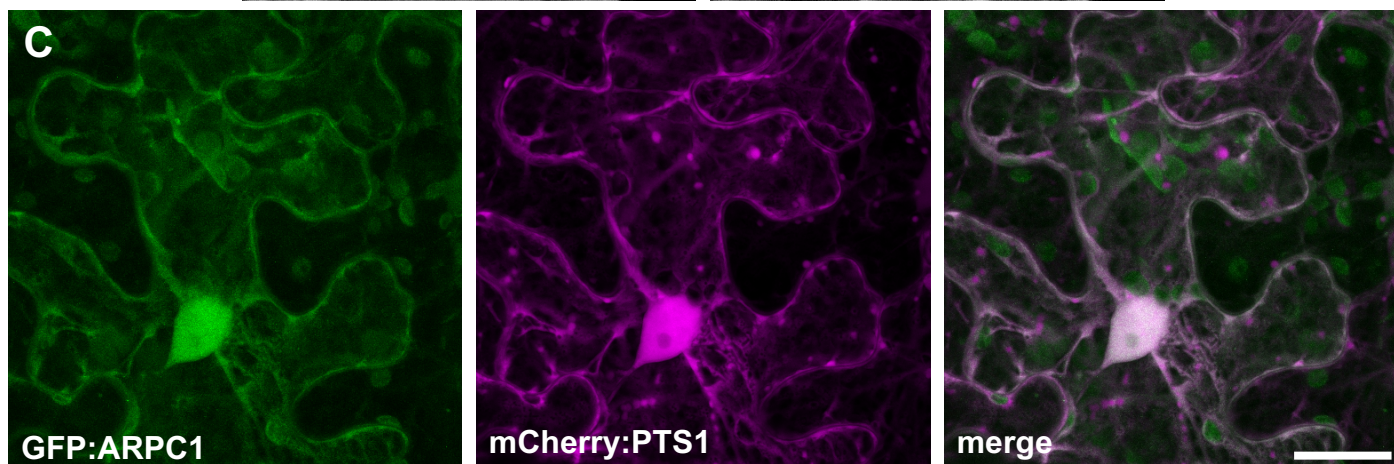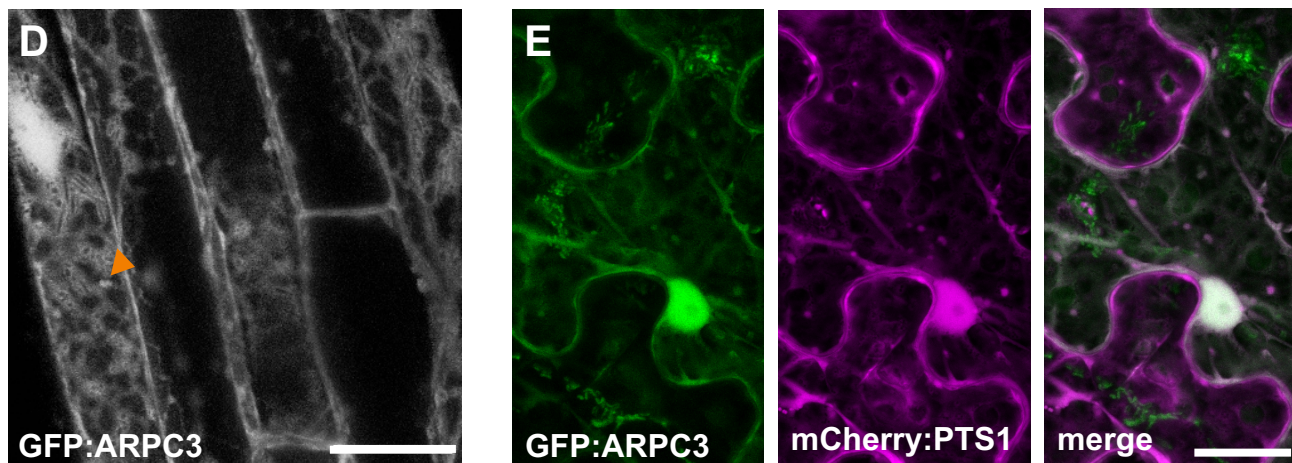

Scalebar = 20 $\mu$ m

Supplement: Supplementary file 7 — Supplementary Information 7. [file 41598_2022_22982_MOESM7_ESM.pdf]
