# Supplementary material for: CRISPR-Cas9 Arabidopsis mutants of genes for ARPC1 and ARPC3 subunits of ARP2/3 complex reveal differential roles of complex subunits
Source: Sci Rep. 2022 Oct 28;12:18205. doi: 10.1038/s41598-022-22982-8 (PMC9616901; doi:10.1038/s41598-022-22982-8)
Supplement: Supplementary file 2 — Supplementary Information 2. [file 41598_2022_22982_MOESM2_ESM.pdf]

CRISPR-Cas9 Arabidopsis mutants of genes for ARPC1 and ARPC3 subunits of ARP2/3 complex reveal differential roles of complex subunits

Authors: Erica Bellinvia, Judith García-González, Petra Cifrová, Jan Martinek, Lenka Sikorová, Lenka Havelková, Kateřina Schwarzerová

Department of Experimental Plant Biology, Faculty of Science, Charles University, Czech Republic

Supplementary information

Supplementary figure 1. Histochemical analysis of *ARPC3* and *ARPC1A* promoter activity.

(A) Promoter activity of the *ARPC3* gene is detected in trichomes.

(B) Analysis of GUS expression driven by the promoter of the *ARPC1A* gene in 6-days-old seedlings expressing the fusions *pARPC1A::GUS*. The seedling cartoon depicts the imaged regions in the histochemical staining experiments.

The expression of the *ARPC1A gene* could be prominently detected in stomatal cells, shoot apical meristem (including developing true leaves), root epidermis, and vascular tissues. In comparison with other subunits, *ARPC1A* expression was less prominent in columella cells and more pronounced in the meristematic region of the stele. Arrowheads indicate detected expression in guard cells.

Supplementary figure 2. Sequence and gene expression analysis of ARPC3 T-DNA insertional lines.

Sequence and gene expression analysis of T-DNA insertional lines obtained from publicly available databases hitting the *ARPC3* gene region. **(A)** Schematic

representation of the three *Arabidopsis thaliana* T-DNA insertional mutants with insertion sites either at the UTR regions (SAIL_131_F01, SALK_099449) or coding region (SAIL_1210_A03_C1) of the *ARPC3* gene. **(B)** qRT-PCR quantification of the *ARPC3* gene expression in the abovementioned lines compared to wild-type.

Supplementary figure 3

Representative image showing the wild-type-like trichomes of the knock-out T-DNA insertional line SAIL_1210_A03-C1.

Supplementary figure 4. **Evaluation of trichome branch number in first true leaves of 14-days-old seedlings.**

Plants lacking functional ARPC1 (*arpc1a/b-c1*) show a distinct phenotype of reduced trichome branching, while in other ARP2/3 complex mutants (*arpc3-c1*, *arpc4,* and *arpc5*) a slight increase in branch number is observed. n=139-607 trichome cells (10-26 plants).

Supplementary figure 5. Subcellular localization of GFP-tagged ARPC1A and ARPC3 subunits.

GFP-ARPC1A was observed to be broadly expressed in the epidermal cells of the root **(A)** meristem and **(B)** elongation zone of stably transformed *arpc1a/b-c1* seedlings. **(C)** GFP-ARPC1 was transiently co-expressed with the peroxisomal marker mCherry-PTS1 in *Nicotiana benthamiana* leaves. **(D)** GFP-ARPC3 was expressed in stably transformed *arpc3-c1* seedlings. Arrow shows occasionally observed dots. Root elongation zone. **(E)** GFP-ARPC3 was transiently co-expressed with the mCherry-PTS1 marker in tobacco leaf epidermis. Co-expression shows occasional colocalization with peroxisomes.

Supplementary figure 6

Complete immunoblots corresponding to the co-immunoprecipitation experiments presented in Figure 6.

Supplementary table 1: list of primers used in this study.

References:

1. Martinek, J. *et al.* ARP2/3 complex associates with peroxisomes to participate in pexophagy in plants. *bioRxiv* 2022.04.07.487451 (2022) doi:10.1101/2022.04.07.487451.
