# Supplementary material for: CRISPR-Cas9 Arabidopsis mutants of genes for ARPC1 and ARPC3 subunits of ARP2/3 complex reveal differential roles of complex subunits
Source: Sci Rep. 2022 Oct 28;12:18205. doi: 10.1038/s41598-022-22982-8 (PMC9616901; doi:10.1038/s41598-022-22982-8)
Supplement: Supplementary file 8 — Supplementary Information 8. [file 41598_2022_22982_MOESM8_ESM.pdf]

Supplementary Table 1

List of Primers

**Primer name**	**Sequence (5' →3')**
qRT-PCR	
ARPC3-F	CTCCTTCCTGATATCGCTTC
ARPC3-R	AAGGTGATTGCCTCGTCTAC
EF1α-F	TGAGCACGCTCTTCTTGCTTTCA
EF1α-R	GGTGGTGGCATCCATCTTGTTACA
UBC9-F	GCTCTCACAATTTCCAAGGTGCTGC
UBC9-R	AGGGTCCTTCCTTAAGGACAGTATTTGTG
Genotyping	
SALK_099449_RP	AGAATCGCCACCTTTAGCTTC
SALK_099449_LP	AAGGTAGAGGCTCAAACGCTC
SAIL_1210_A03C1_RP	CGGTACGAAGCAATGTACCAC
SAIL_1210_A03C1_LP	GAAGCCAAACAGAACACAAGG
SAIL_1210_A03_RP	AGCAAGCAAAGCATTCTTCTG
SAIL_1210_A03 LP	GTTGTTATCAGAGAGCCGCAG
SAIL_131_F01_RP	GGTGATCCGTACCATTTTGTG
SAIL_131_F01_LP	ACAATGACCAGAGCCATAACG
Amplification of the gRNA template in the CRISPR protocol	
DT1-BsF_arpC1_1	ATATATGGTCTCGATTGGACATGTAACCTCTCCCAGGTT
DT1-F0_arpC1_1	TGGACATGTAACCTCTCCCAGGTTTTAGAGCTAGAAATAGC
DT2-R0arpC1_1	AACTTGGACACAAAGTGCAGCACAATCTCTTAGTCGACTCTAC
DT2-BsRarpC1_1	ATTATTGGTCTCGAAACTTGGACACAAAGTGCAGCACAA
DT1-BsF_arpC3_1	ATATATGGTCTCGATTGTGGCGTTAAAGAGGCTTGAGTT
DT1-F0_arpC3_1	TGTGGCGTTAAAGAGGCTTGAGTTTTAGAGCTAGAAATAGC
DT2-R0arpC3_1	AACGTTGGCTCTATATGCTACACAATCTCTTAGTCGACTCTAC
DT2-BsRarpC3_1	ATTATTGGTCTCGAAACGTTGGCTCTATATGCTACACAA
CRISPR target sequence amplification	
ARPC1 seq F	CTCTGTCCTAACAACACTG
ARPC1 seq R	TAGCACCACTTCCAAACAG
ARPC3 seq F	GTGCTAATGTCTTCTTCAC
ARPC3 seq R	AGTGGCTAACACCTATGC
Cloning in pGreen and sequencing	
ARPC1 F	GGTCCAATGGCAGTGGTGGTGGATGTTCATC

ARPC1 R	AAGCTTTTAAAACTGATTGCCAAGTTCTTG
ARPC3 F	GGTCCAATGGGCAGTGGTGGATGTTCATC
ARPC3 R	AAGCTTTCATAGGACGACCACGTTCATGAAT
GFP F	GGATCCAAGGAGATATAACAATGGTGAGCAAGGGCGAG
ARPC1 promoter	
pARPC1A-F	AAGCTTTCTTCAACTCTTGGCATTGGTG
pARPC1A-R	GGATCCTCTTTTCTCTCAAATTGACATCCAC
Yeast complementation	
BamHI-ARPC3-F	AAAAAAGGATCCAATGGTTTATCACTCGAGTTTTGTG
HindIII-ARPC3-R	TTTTTTAAGCTTTCATAGGACGACCACGTTCATGAAT
PstI-ARPC3-R	TTTTTTCTGCAGTCATAGGACGACCACGTTCATGAAT
